# Genetic Variability and Phylogeny of Current Chinese Porcine Epidemic Diarrhea Virus Strains Based on *Spike*, *ORF3*, and *Membrane* Genes

**DOI:** 10.1155/2014/208439

**Published:** 2014-01-22

**Authors:** Ruiqin Sun, Zhangming Leng, Shao-Lun Zhai, Dekun Chen, Changxu Song

**Affiliations:** ^1^College of Veterinary Medicine, Northwest Agriculture and Forestry University, Xinong Road, Yangling District, Xianyang 712100, China; ^2^Institute of Animal Health, Guangdong Academy of Agricultural Sciences, Baishigang Street, Wushan Road, Tianhe District, Guangzhou 510640, China; ^3^College of Veterinary Medicine, South China Agricultural University, Guangzhou 510642, China

## Abstract

Since late 2010, the outbreak of porcine epidemic diarrhea (PED) in China has resulted in the deaths of millions of suckling piglets. The main cause of the disease outbreak was unknown. In this study, partial *spike* (*S*), *ORF3*, and *membrane* (*M*) genes amplified from these variants were sequenced and analyzed. The results showed that the variants could be clustered into one to three subgroups and suggested that *S* genes were variable, while *M* genes were relatively conserved. Moreover, in comparison with the vaccine strain CV777, sequence alignment analyses revealed that the *S* genes of the newly isolated strains contained several mutations at the aa level. It is possible that these mutations have changed the hydrophobicity of the S protein and influenced the viral antigenicity and virulence. Interestingly, homology analyses based on ORF3 demonstrated that the isolates had an intact opening reading frame (ORF), which were different from the attenuated DR13 strain. In conclusion, the widespread PED virus (PEDV) isolates had virulent characteristics. Additionally, the high degree of variation in the genes, particularly *S* genes, might provide an explanation for the poor immunity and rapid spread of the disease.

## 1. Introduction

PED, caused by PEDV, is a highly contagious, enteric disease of swine. The main symptoms are vomiting, dehydration, and a high mortality in piglets [1]. PED is a serious health problem in suckling and nursing pigs that are typically 1-2 weeks of age. The mortality rate can be as high as 100% in 10-day-old or younger piglets. Pigs of all ages are susceptible to PEDV infection [2, 3]. PED occurrence has been reported in many countries, including Japan, Korea, France, Belgium, and Switzerland [4–6]; it was first reported in China in 1976 [7]. Since late 2010, millions of piglets have been killed in China during PED outbreaks, which significantly affected the pork meat supply and price [8]. PED becomes a severe disease and has led to a significant loss in the swine industry.

PEDV has a positive-sense single-stranded RNA that encodes major proteins, including *spike* (*S*), *membrane* (*M*), small membrane (sM), nucleocapsid (N), and envelope (E), translated from its subgenomic mRNA [9]. The S protein of PEDV is an important site of viral neutralization [10, 11]. The M protein, that is, structural M glycoprotein, plays an important role in the assembly process of the viral nucleocapsid and membrane [12]. Previous studies had revealed that the 624-nucleotide (nt) *ORF3* in attenuated-type PEDVs contains 51 nt deletions compared to wild-type PEDVs, and this deletion region might be important for PEDV pathogenicity [13, 14].

The commercially produced vaccines, that is, inactivated transmissible gastroenteritis (TGEV H) and porcine epidemic diarrhea (CV777), were used to protect swine from the PED and TGE diseases in China. Despite the administration of the vaccine, PED still affected farms in many provinces in late 2010 [8]. The reason for this remains unknown. To understand the genetic variations of PEDV and explain the remaining instances of PEDV, PEDV RNA was extracted directly from nasal and rectal samples of naturally infected piglets. The partial *S*, *ORF3*, *M* genes were amplified, sequenced, and analyzed. The amino acid (aa) sequences were predicted, based on which the phylogenetic trees were constructed and the hydrophobicity was analyzed. The similarities and differences between current and reference PEDVs were elucidated. These analyses were also useful for further molecular biology studies of PEDV strains that were prevalent in China.

## 2. Materials and Methods

### 2.1. Samples

A total of 70 rectal swab samples were collected from 70 piglets infected with PEDV from October 2010 to March 2012. These infected piglets were chosen from 16 different swine farms located in 6 different provinces in China. Samples were subgrouped according to the geographic regions (Table 1). The rectal swabs were soaked in PBS buffer supplemented with penicillin G (10,000 IU/mL) and streptomycin (2 mg/mL) and centrifuged at 1,500 ×g for 15 min to collect the supernatant fluids. The supernatant fluids were stored at –80°C until used.

### 2.2. RNA Extraction

RNA was extracted from the supernatant using TRIzol Reagent (Invitrogen Corp., Carlsbad, CA, USA) following the manufacturer's instructions. Briefly, the supernatant (200 *μ*L) containing PEDV was mixed with 1 mL of TRIzol Reagent. Then, 200 *μ*L of chloroform was added to the mixture, and the suspension was centrifuged for 15 min at 12,000 ×g. The RNA-containing aqueous phase was precipitated with the same volume isopropanol, maintained at –20°C for 1 h, and centrifuged for 10 min at 12,000 ×g. The resulted RNA pellet was washed with 1 mL of 75% ethanol, centrifuged for 10 min at 12,000 ×g and dried, and resuspended in 30 *μ*L of diethyl-pyrocarbonate- (DEPC-) treated deionized water.

### 2.3. Primers

Pairs of sense and antisense primers were designed and aligned based on the nt sequence of the *S, M, ORF3* genes from the GenBank database (National Center for Biotechnology Information, USA). These primers were used to amplify the PEDV gene of interest. The primer sequences and other details are shown in Table 2.

### 2.4. RT-PCR and Sequencing

RT-PCR was performed using PrimeScript One Step RT-PCR Kit Ver. 2 (TaKaRa Biotechnology Co., Ltd., Dalian, China). Primers S-F and S-R, ORF3-F and ORF3-R, and M-F and M-R, as shown in Table 2, were used to amplify the corresponding partial S, ORF3, and M genes from PEDV, respectively. For RT-PCR, 3 *μ*L of extracted viral RNA was mixed with a reaction mixture containing 25 *μ*L 2 × 1 Step Buffer, 2 *μ*L each specific primer (10 pmol), 2 *μ*L PrimeScript 1 Step Enzyme Mix, and 16 *μ*L RNase Free water. The amplifications for the *S*, *ORF3* genes were performed with reverse transcription at 50°C for 30 min and predenaturation at 95°C for 5 min, followed by 35 cycles of denaturation at 95°C for 1 min, annealing at 50°C for 1 min, and extension at 72°C for 1 min, and a final extension step of 72°C for 10 min. For the *M* gene, the amplification conditions were the same as above; however, the annealing temperature used was 56°C for 1 min. RT-PCR products were visualized via electrophoresis in a 1.5% agarose gel containing ethidium bromide. Bands of the correct size were excised and purified using TaKaRa MiniBEST Agarose Gel DNA Extraction Kit Ver. 3.0 (TaKaRa Biotechnology Co., Ltd., Dalian, China) following the manufacturer's instructions. The purified products were sequenced by BGI Biological Engineering Technology & Services Co., Ltd. (China, Shenzhen).

### 2.5. Sequence Analysis

Nucleotide and deduced aa sequences were analyzed using the CLUSTALX v1.83, Bioedit v7.0.5.2 programs, and MegAlign software for alignment and sequence analysis. *S*, *M*, *ORF3* nt and deduced aa sequences were compared with the PEDV reference strains listed in Table 2. The resulting subsets were edited manually.

### 2.6. Phylogenetic Analysis

Using a neighbor-joining (NJ) method in MEGA version 5.1, phylogenetic trees were generated using an alignment of *S*, *M*, *ORF3* gene nt and the aa sequences deduced from the reference PEDVs. To assess the relative support for each clade, bootstrap values were calculated from 1,000 analysis replicates, and the cut-off point for bootstrap replication was 50%.

## 3. Results

### 3.1. Amplification and Sequencing of *S*, *ORF3*, and *M* Genes

To obtain the nt sequence, the partial *S*, *ORF3*, *M* genes were amplified in PED positive samples using one-step RT-PCR. The predicted 651-bp, 833-bp, and 808-bp bands for *S*, *ORF3*, *M* genes, respectively, were detected in the resulted PCR products. Subsequently, these products were purified and sequenced.

### 3.2. *S* Gene Analysis

The sequence alignment results showed that the sequence homogeneity between the reference isolate and the newly isolated variants (88.7–97.3% for nt and 85.2–98.0% for aa) was lower than that observed within the newly isolated strains alone (96.7–99.8% for nt and 94.6–100% for aa). These newly isolated strains had nt homology of 94.6–97.3%, 88.6–91.1%, 92.8–95.5%, and aa homology of 94.4–98.0%, 85.2–88.8%, 91.4–95.4% when comparing to CV777, Chinju99, and CH/S sequences (data not shown).

**Figure 1:**
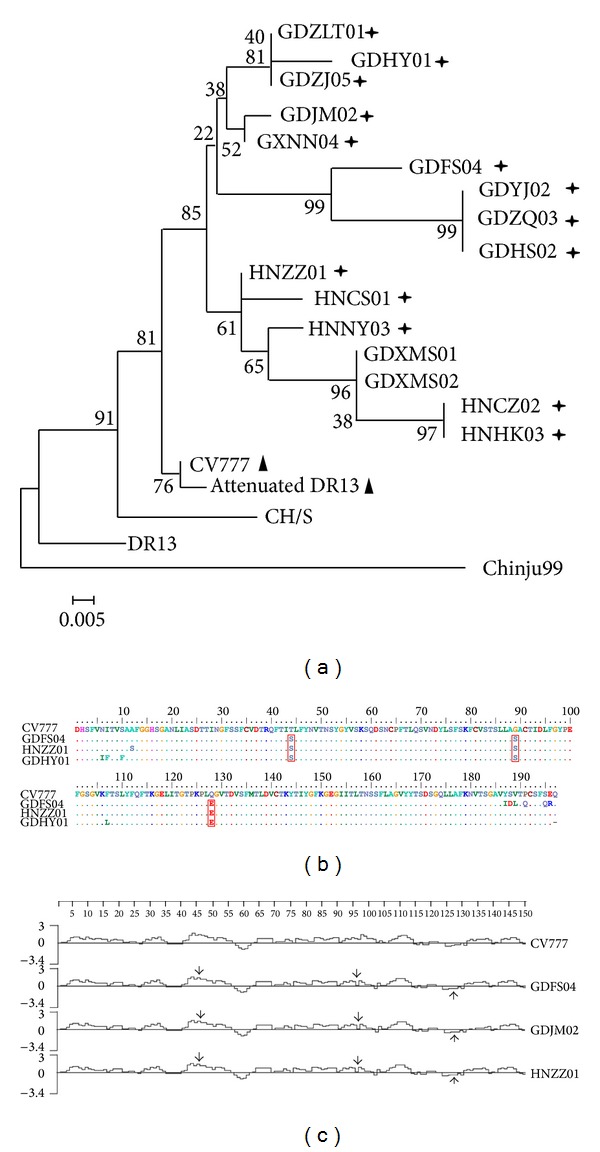
Comprehensive analysis of *S* genes from current PEDV with reference strains. (a) Phylogenetic analysis based on S aa; (b) point mutations analysis of S aa; (c) hydrophobic analysis of S aa. Phylogenetic tree is constructed by the neighbor-joining method with the MEGA 4.0 program. The percentage bootstrap support (per 1000 replicates) is indicated by the values at each node. Cross asterisk indicated present isolates; triangle indicated attenuated strains; box indicated point mutations.

A phylogenetic tree was generated based on the deduced aa sequences of the partial *S* gene. In all phylogenies, the reference aa sequences, observed in samples collected from previous outbreaks, tended to cluster near the root but had a poorly resolved branching structure. This was likely due to relatively low sequence divergence. In contrast, results analyzed using NJ method with bootstrap support indicated that viruses from more recent infections formed three well-supported lineages that tended to cluster far from the root. As expected. These lineages were denoted as groups I, II, and III in the S phylogenies (Figure 1(a)).

To further investigate the nt and deduced aa substitutions, partial S sequences amplified from 16 isolates were sequenced and sorted according to the collection date and district. The fragments were aligned at position 1523–2115 of the complete *S* gene using Blast (http://blast.ncbi.nlm.nih.gov/Blast.cgi). In this study, three aa mutations were detected at positions 44 (T → S), 89 (G → S), and 128 (Q → E) (Figure 1(b)). Sun et al. [15, 16] suggested that this region might be considered an important immunodominant domain for PEDV. The hydrophobicity of the deduced aa for these newly isolated strains was then analyzed, and the mutation impact on the hydrophobicity of S protein was confirmed (Figure 1(c)).

### 3.3. *ORF3* Gene Analysis

The nt sequence alignment results showed that the sequence homogeneity among the newly isolated strains (96.1–100%) was higher than that observed between the attenuated-type PEDVs and the newly isolated strains (89.6–98.8%). Likewise, the aa sequence alignment results showed a similar pattern (i.e., 96.7–100% among the current isolates and 91.1–100% between the attenuated-type PEDVs and the current isolates). More precisely, the newly isolated strain DR13 showed higher homology (97.2–98.7% for nt and 96.7–99.4% for aa) than both attenuated DR13 (89.6–90.7% for nt and 91.1–93.3% for aa) and CV777 (89.9–91% for nt and 91.1–93.3% for aa) (data not shown).

**Figure 2:**
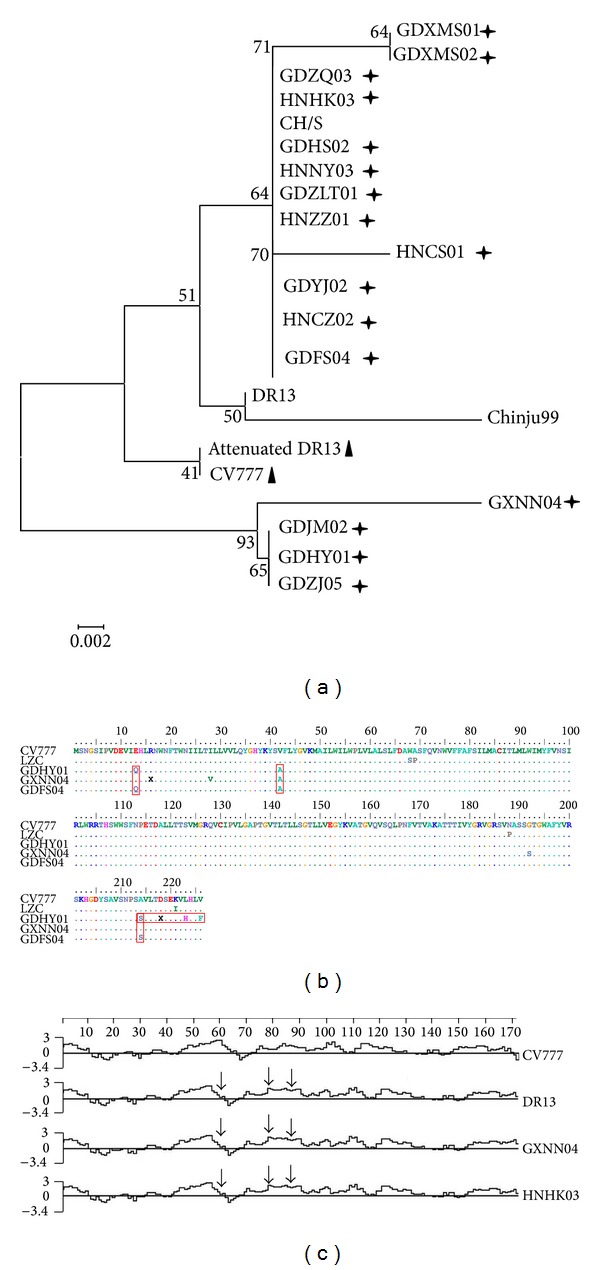
Comprehensive analysis of *ORF3* genes from current PEDV with reference strains. (a) Phylogenetic analysis based on ORF3 aa; (b) point mutations analysis of ORF3 aa; (c) hydrophobic analysis of S aa. Phylogenetic tree is constructed by the neighbor-joining method with the MEGA 4.0 program. The percentage bootstrap support (per 1000 replicates) is indicated by the values at each node. Cross asterisk indicated present isolates; triangle indicated attenuated strains; box indicated point mutations.

A phylogenic tree was constructed using the ORF3 aa sequences from the newly isolated strains and the references isolates. All of the sequences were divided into four phylogenetic groups: group I contained current isolates and reference strain CH/S, group II contained reference strains DR13 and Chinju99, group III contained attenuated DR13 and CV777, and group IV current isolated strains GXNN04, GDJM02, GDHY01, and GDZJ05. Further analysis showed that the current isolated groups (I and IV) were independent of the reference strain groups (II and III) (Figure 2(a)).

Sequence analysis of the complete *ORF3* genes showed that all of the newly isolated strains had intact ORFs that could regulate the translation of a 224 aa residues. Nevertheless, there was a large deletion region, approximately 51/49 nt (at positions 245–295/293), in the attenuated DR13 and CV777 PEDV, which accounted for the missing of 17/16 aa (YCPLLYYCGAFLDATI/I, at positions 83–98/97) (Figure 2(b)). The mutations observed at aa positions 7 (Q → X), 80 (V → F), and 182 (H → Q/Y) did not impact hydrophobicity (Figure 2(c)).

### 3.4. *M* Gene Analysis

Based on the complete *M* gene sequence, the newly isolated strains showed 98.5–100% nt sequence homogeneity within the group but 96.5–98.1% and 97.2–98.2% when compared to those stains previously isolated in China (LZC, CH/S, and LJB/03) and those foreign isolates (CV777 and Chinju99), respectively. Meanwhile, the newly isolated strains showed 97.8–100% aa sequence homogeneity within the group and 95.6–98.7% and 97.3–98.7% when compared to those previously isolated Chinese strains and foreign strains, respectively.

**Figure 3:**
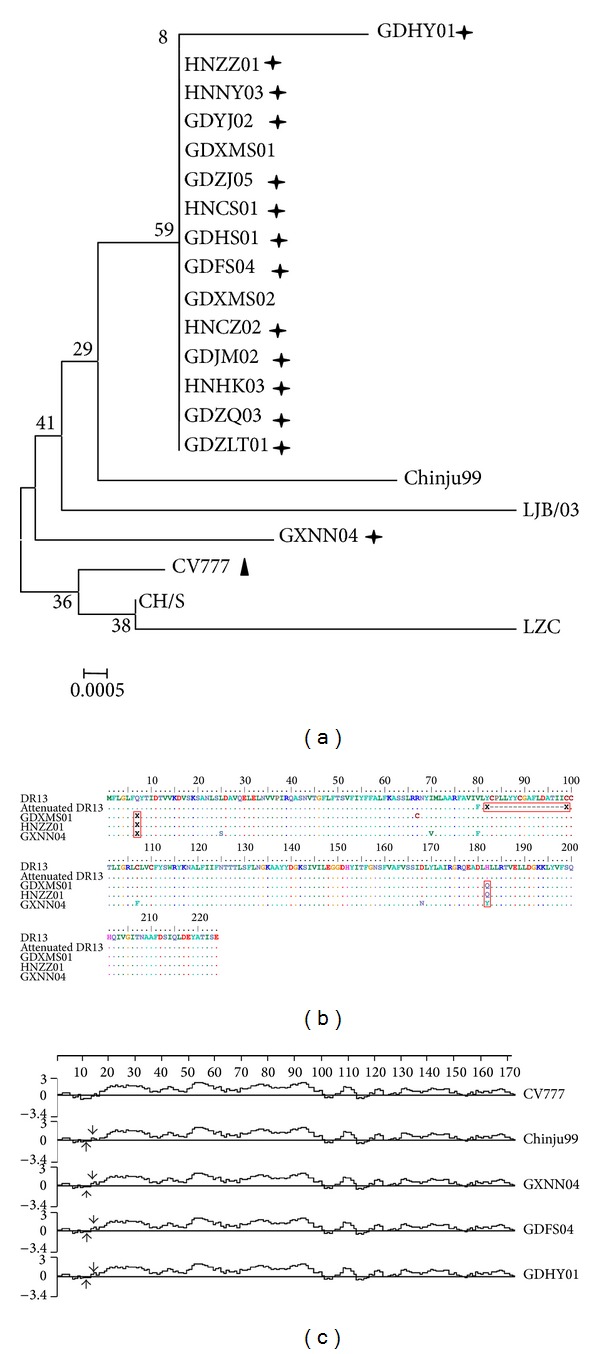
Comprehensive analysis of *M* genes from current PEDV with reference strains. (a) Phylogenetic analysis based on M aa; (b) point mutations analysis of M aa; (c) hydrophobic analysis of S aa. Phylogenetic tree is constructed by the neighbor-joining method with the MEGA 4.0 program. The percentage bootstrap support (per 1000 replicates) is indicated by the values at each node. Cross asterisk indicated present isolates; triangle indicated attenuated strains; box indicated point mutations.

A phylogenetic tree was generated based on the deduced aa sequence of the *M* gene. With the exception of the GXNN04 strain, all of the newly isolated strains were placed into one subgroup. The other subgroup contained vaccine strains CV777 and early Chinese strains CH/S and LZC. It is noted that the GXNN04 isolate was closer to the Korean strains Chinju99 and LJB/03 than the other newly isolated strains (Figure 3(a)).

All PEDV field strains isolated during the recent outbreaks had a single ORF of 681 nt, which encoded a protein of 227 aa. Except for several point mutations, there was no deletion or insertion within the ORFs of these isolates. The isolates had a conserved intergenic motif (ATAAAC) 5 nt upstream of the initiating ATG, which was previously recognized in Br1/87, CV777, LZC, and QH [17]. Compared to PEDV CV777, the new isolates had 11 nt mutations leading to 3 aa mutations. It is noted that the aa mutations at position 13 (E → Q) altered the hydrophobicity of the N terminus of the M protein (Figure 3(b)) and therefore had impact on its antigenic activity, whilst the other aa mutations, such as those at position 42 (V → A) and 214 (A → S), did not influence hydrophobicity of the M protein (Figure 3(c)).

## 4. Discussion

Gene sequencing analyses are useful tools to help us understand viral variation and pathogenicity. Several genes in PEDV have been analyzed previously, such as the *N*, *S* genes of PEDV Chinju99 isolated in 1999 in Korea, the *M* gene of PEDV CH/IMT/06 isolated in China, and the *ORF3* gene of PEDV DR13 and attenuated DR13 [13, 17, 18]. More recently, some PEDV variants isolated in China (2010 to 2012) have also been reported [19]. However a comprehensive analysis to compare these three genes (i.e., partial *S*, *ORF3*, and *M*) has never been reported. In this study, partial *S*, *ORF3*, *M* genes from 16 PEDV strains isolated from the current Chinese PEDV outbreak were amplified and sequenced. The sequencing results were used to determine the genetic diversities and molecular characterizations of all of the PEDV strains.

The S protein of PEDV is exposed directly to the immune system of the host and therefore has always been used as a marker of viral variation. In this study, the data demonstrated that there was significant variation in *S* genes between the attenuated and virulent strains. Previous studies corroborated these results [8, 19]. Additionally, the newly isolated PEDV strains were highly homologous with each other but less similar to the vaccine strain CV777 or the virulent strain Chinju99 from Korea and CH/S from previous Chinese outbreaks. In the phylogenic trees, the current strains from different districts were genetically divided into different subgroups, but within each subgroup, the strains did not share the same geographical features. Also, these subgroups were genetically different from the groups of both attenuated strains (i.e., CV777 and attenuated DR13) and previous virulent strains (i.e., DR13, CH/S, and Chinju99). We can therefore draw a conclusion that the current strains in China represent a distinctive PEDV group and it is stable and virulent. The current strains displayed significant genetic variation from both the vaccine strains and previous strains. This may provide one possible answer for the nationwide outbreak and the poor efficacy of the vaccines. Previous studies showed that the S glycoprotein played an important role in viral attachment and penetration and was the neutralizing antibodies-inducing region due to the 2 B-cell linear epitopes [15]. In this study, the partial S proteins of current isolates, containing different aa to the previous strains due to mutations, were analyzed to determine the possible change on the protein hydrophobicity. When compared to the vaccine strain CV777, the current strains showed a significant hydrophobic alternation. This change was induced by a 4–9 aa mutations in the neutralizing epitope region of the current isolates and could provide one explanation for the poor immunity.

*ORF3*, located between the *S* and *E* genes, was recognized as a marker for the attenuated and virulent strains through an analysis of attenuated DR13 and wild-type DR13. These studies suggested that this area of the genome may be involved in cell tropism and the pathogenicity of the virus [13, 20, 21]. Park et al. [10] analyzed cell-culture-adapted PEDV (passage 100) with a smaller *ORF3* gene and reduced virulence from the wild-type virus. In this study, the PEDV strains isolated from the current Chinese outbreaks had intact *ORF3* genes that encoded 225 aa, which was in accordance with a previous study [13]. The phylogenic tree analysis demonstrated that the current strains should be clustered into the same group, despite of the different temporal and geographical origins (Figure 2(a)). This suggested that the ORF3 sequence was relatively conserved among stains from different temporal and geographical origins. A hydrophobicity analysis indicated that current isolates were different from the attenuated PEDV DR13 and vaccine strain CV777, which might be due to their adaptability and viral replication [14].

The 226 aa M protein of PEDV was proved to play an important role in viral package and budding. The current isolates shared 98.5–100% and 97.8–100% similarity in their nt and aa sequences, respectively. The phylogenetic tree suggested that all of the present PEDV strains, except for GXNN04, should be clustered into one subgroup, which demonstrated that the *M* gene was relatively conserved. This conclusion was consistent with the results from an earlier study [17]. A hydrophobicity analysis indicated there was only one mutation at aa 13 (E → Q) that would have affected the hydrophobicity of the M protein; however, the function of this mutation requires further investigation.

This study investigated the homology, phylogeny, and hydrophobicity of the partial *S*, full *ORF3*, and *M* genes from 16 currently prevalent PEDVs in China. The results suggested that most of the strains studied are highly homologous to each other, despite being clustered into different subgroups based on the *S*, *M*, and *ORF3* genes. However, all of the current strains were distant from the vaccine strains genetically based on the *S*, *M*, and *ORF3* genes analysis. The above analysis would help us to understand the origin of the virus and provided a reasonable explanation for both the outbreak and the poor efficacy of the commercial vaccine.

## Figures and Tables

**Table 1:** Detail information of present and reference sequences used in Figures 1(a), 2(a), and 3(a) including country/district and collection date.

Country/District	Strain	Collection date
Nanning, Guangxi	GXNN04	Nov-2010
Heyuan, Guangdong	GDHY01	Dec-2010
Jiangmen, Guangdong	GDJM02	Feb-2011
Zhanjiang, Guangdong	GDZJ05	Mar-2011
Nanyang, Henan	HNNY03	Apr-2011
Zhengzhou, Henan	HNZZ01	May-2011
Cenzhou, Hunan	HNCZ02	May-2011
Haikou, Hainan	HNHK03	Jun-2011
Zhongluotan, Guangdong	GDZLT01	Jul-2011
Zhaoqing, Guangdong	GDZQ03	Sep-2011
Foshan, Guangdong	GDFS04	Oct-2011
Yangjiang, Guangdong	GDYJ02	Dec-2011
Heshan, Guangdong	GDHS02	Mar-2012
Changsha, Hunan	HNCS01	Feb-2012
Guangzhou, Guangdong	GDXMS01	Mar-2012
Guangzhou, Guangdong	GDXMS02	Mar-2012
Korea	Chinju99	1999
Lanzhou (China)	LZC	Dec-2006
Heilongjiang (China)	LJB/03	2003
Belgium	CV777	1977
Korea	DR13	unknown
Korea	Attenuated DR13	unknown
China	CH/S	1986

**Table 2:** Primers used in RT-PCR for PEDV spike, ORF3, and membrane genes.

Primer	Nucleotide sequence (5′-3′)	Size of product (bp)
S-F	TTCTGAGTCACGAACAGCCA	651
S-R	CATATGCAGCCTGCTCTGAA	
ORF3-F	TCCTAGACTTCAACCTTACG	833
ORF3-R	GGTGACAAGTGAAGCACAGA	
M-F	GTCTTACATGCGAATTGACC	808
M-R	GGCATAGAGAGATAATGGCA	
